# Effects of low-intensity pulsed ultrasound on recovery of physical impairments, functional performance and quality of life after total knee arthroplasty: Protocol for a quasi-experimental study

**DOI:** 10.1097/MD.0000000000017045

**Published:** 2019-09-06

**Authors:** Munayati Munajat, Nor Azlin Mohd Nordin, Nor Hamdan Mohamad Yahya, Ahmad Hafiz Zulkifly

**Affiliations:** a Center for Rehabilitation and Special Needs, Faculty of Health Sciences, Universiti Kebangsaan Malaysia, Kuala Lumpur; b Department of Physical Rehabilitation Sciences, Faculty of Allied Health Sciences, International Islamic University Malaysia, Kuantan, Pahang; c Department of Orthopedics and Traumatology, Faculty of Medicine, Universiti Kebangsaan Malaysia, Kuala Lumpur; d Department of Orthopedics, Traumatology and Rehabilitation, International Islamic University Malaysia Medical Centre, Kuantan, Pahang, Malaysia.

**Keywords:** low-intensity pulsed ultrasound, physiotherapy, total knee arthroplasty

## Abstract

**Introduction:**

The presence of significant pain and swelling during the acute stage following total knee arthroplasty (TKA) may limit the patients’ ability to cooperate in intensive physiotherapy interventions. Low-intensity pulsed ultrasound is one of the modalities that can be used for acute pain and swelling management. However, only one study investigated the effect of this modality in patients with TKA. There is limited documentation of the effects of combining low-intensity pulsed ultrasound in TKA rehabilitation in the recovery of physical impairments and how these influence the recovery of function after TKA. Therefore, this study is proposed with the aim to evaluate the effects of low-intensity pulsed ultrasound as an adjunct to conventional physiotherapy on the recovery of physical impairments, functional performance and quality of life after TKA surgery.

**Methods:**

This is an assessor-blinded quasi-experimental study comparing two approaches of physiotherapy, namely pulsed ultrasound-added physiotherapy and conventional physiotherapy. Total number of participants with TKA required for this study will be calculated based on the result of a pilot study. Participants will be alternately allocated into either pulsed ultrasound-added physiotherapy group (low-intensity pulsed ultrasound and conventional physiotherapy) or control group (conventional physiotherapy). Pulsed ultrasound-added physiotherapy group will receive low-intensity pulsed ultrasound starting at post-operative day 2 (4–5 times for the first-week after surgery and 2–3 times a week for a further 2 weeks). Both groups will receive conventional physiotherapy 4 to 5 times for the first-week after surgery and 2 to 3 times a week for a further 11 weeks. This procedure and process will be tested and established in a pilot study. Primary outcomes of interest are pain level, swelling, active range of knee motion, and quadriceps strength. The secondary outcomes are functional performance and quality of life.

**Discussion:**

This study will fill the gaps in knowledge relating the benefits of including low-intensity pulsed ultrasound into conventional physiotherapy for patients with TKA.

**Trial registration:**

Australian New Zealand Clinical Trials Registry, ACTRN12618001226291

## 1 Introduction

In Malaysia, Community-Oriented Program for the Control of Rheumatic Diseases (COPCORD) survey found that 15.4% of 2594 Malaysians were diagnosed with knee osteoarthritis (OA) and more than half of 64.8% respondents with knee pain had OA-related knee pain.^[1]^ The high incidence of knee OA has been reported in countries around the world.^[2–4]^

Patients with knee OA may receive electrotherapy,^[5,6]^ joint mobilization,^[7]^ therapeutic exercises,^[8]^ virtual reality-based therapy,^[9]^ or intra-articular injection of hyaluronic acid^[10]^ to reduce pain and/or to improve functional ability. However, total knee arthroplasty (TKA) is recommended as an intervention for managing severe knee OA^[11]^ or when the conservative treatments have failed.^[12]^ The TKA followed by post-operative rehabilitation interventions namely exercises, education, dietary advice, use of insoles, and pain medication has been shown as beneficial to reduce pain and improve function of patients with moderate-to-severe knee OA.^[13]^ The patients after TKA demonstrated excellent and good early functional outcomes.^[14]^

Previous studies have shown that the recovery of physical impairments after TKA which consist of pain,^[15–17]^ knee swelling,^[18,19]^ limitation of knee range of motion,^[15,19,20]^ and quadriceps weakness^[16,19,20]^ varies. Pain^[17]^ and swelling^[18,21]^ after TKA were shown to lead to the development of quadriceps weakness and limitation of knee range of motion.^[20]^ These problems can result in reduced functional performance.^[18,21,22]^ The pain experience and decreased knee mobility after TKA may further weaken the muscles and prevent the patients from being independent in functional activities.^[17]^ A study stated that pain control at early stage of acute recovery is important to improve physical function after TKA.^[23]^ Thus, it is essential to address acute post-operative pain and swelling in order to optimize effectiveness of other interventions and improve functional recovery after TKA.

Past studies had explored the effects of adjunct modalities such as cryotherapy^[24,25]^ transcutaneous electrical nerve stimulation (TENS)^[26]^ and music therapy^[27,28]^ on recovery from pain following TKA. Cryotherapy is a common method used for patients with TKA in reducing acute post-operative pain and swelling. However, the evidence on the effects of cryotherapy remains inconclusive. Markert^[24]^ reported in general evidence shown that cryotherapy did not significantly help to reduce knee swelling, decrease in blood loss and improve range of motion of the operative knee. However, the researcher believed that cryotherapy is still useful for patients with TKA; there is evidence that the cold and compression effect provide positive outcomes. Besides, a review study^[29]^ reported that although some benefits have been shown from the use of cryotherapy following TKA, it is still inconclusive due to the limited number of Level 1 studies to support the use of cryotherapy. Another review study^[25]^ reported that most of previous studies showed positive findings on the use of cryotherapy after knee arthroplasty such as reduced pain and the use of analgesic, and improved knee range of motion and patients’ satisfaction. The study also recommended an application of cryotherapy for knee arthroplasty patients. Despite this, the researchers still highlighted that several studies did not show a significant positive effect of the application of cryotherapy.

Recently, another modality, namely pulsed ultrasound with low-intensity has been found useful in the acute management of patients with TKA. The therapy was also not stated as a contraindication for the patient with metal implants.^[30]^ However to date, this finding was reported only in one study.^[31]^ The researchers^[31]^ investigated the effect of combining low-intensity pulsed ultrasound with cryotherapy on recovery of joint function and the inflammatory index C-reactive protein in female patients with TKA. No adverse effects were reported, indicating that the modality is safe to be used on patients with TKA. The researchers found that patients who received low-intensity pulsed ultrasound in combination with cryotherapy shown greater improvement in the recovery of joint function, knee range of motion and reduction of inflammation compared to patients who received cryotherapy or low-intensity pulsed ultrasound alone. However, the study did not look into the effect of the adjunct modality on the recovery of all physical impairments which also include pain, swelling and quadriceps weakness during early stage post-TKA and how these influence the recovery of function in the post-acute phases. Further resources for acute management after TKA are needed to improve acute pain control^[32]^ and future research on the effect of impairment-targeted interventions in acute hospital and inpatient rehabilitation setting is recommended.^[33]^

The objective of this quasi-experimental assessor-blinded study is to evaluate the effects of low-intensity pulsed ultrasound as an adjunct to conventional physiotherapy on the recovery of physical impairments, functional performance and quality of life in patients with TKA. This study also intends to assess the influence of the recovery of physical impairments following Pulsed ultrasound-added physiotherapy on functional performance and quality of life of patients with TKA.

## 2 Methods

### 2.1 Study design and setting

**Figure 1:**
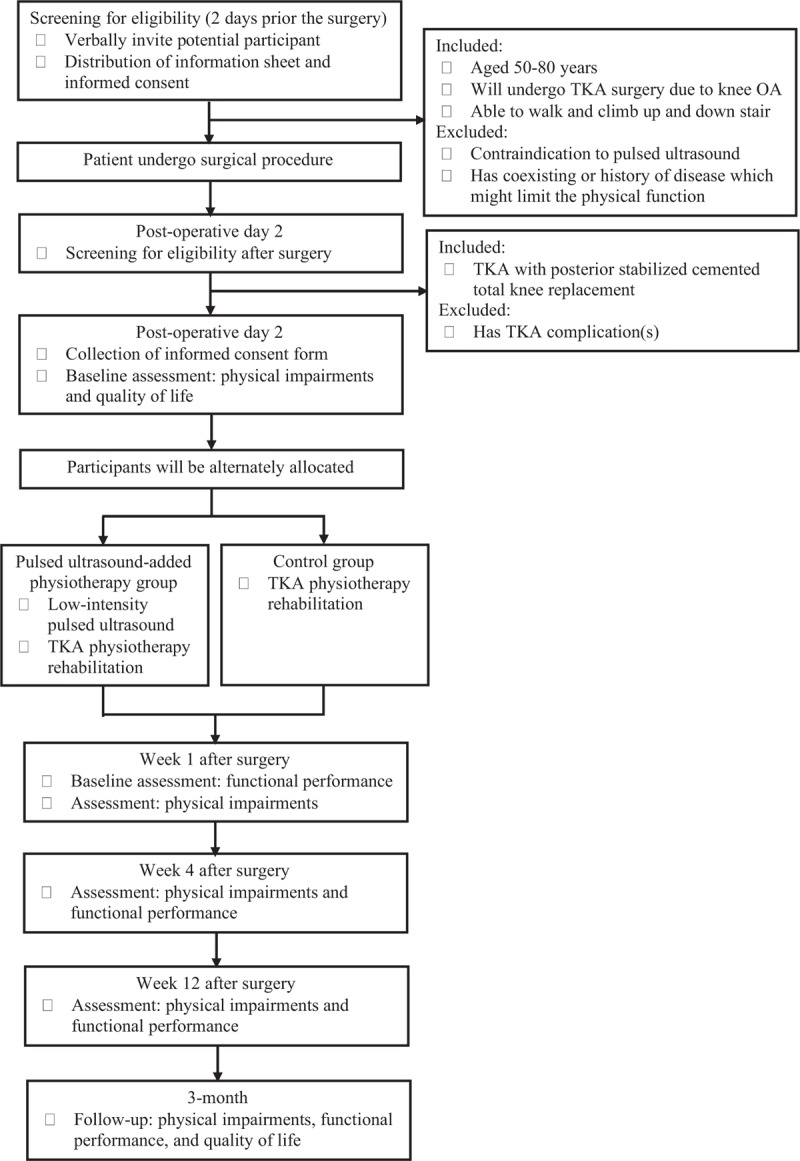
Flow chart of the study. TKA = total knee arthroplasty, OA = osteoarthritis.

This is a quasi-experimental assessor-blinded study comparing two approaches of physiotherapy, namely Pulsed ultrasound-added physiotherapy group or control group with the participant's allocation ratio is 1:1. This study will be conducted at orthopedic wards and physiotherapy department in the Universiti Kebangsaan Malaysia Medical Centre, Kuala Lumpur, Malaysia. The flow of the study is shown in Figure 1. The process in this study will be first tested in a pilot study, following which a main study will be conducted.

### 2.2 Study participants

Inclusion criteria are as follows:

1. Aged 50 to 80 years.
2. Patients who are listed for TKA surgery due to knee OA.
3. The TKA with posterior stabilized cemented total knee replacement.
4. Patients who reported able to walk and climb up and down stairs with or without walking aids.

Exclusion criteria are as follows:

1. Any contraindications to pulsed ultrasound (e.g., malignancy at the tissue to be treated, bleeding at the area of the surgery and deep vein thrombosis).
2. Coexistence or history of diseases which might limit the physical function and functional performance such as old fractures in the lower limb, neurology conditions (e.g., stroke), and other musculoskeletal problems.
3. Has TKA complication(s) such as myocardial infarction, deep vein thrombosis, septic arthritis and neurological deficit due to regional anesthesia complications.

### 2.3 Participants allocation

A systematic allocation method is used to allocate participants alternately into either pulsed ultrasound-added physiotherapy group or control group. The first participant is allocated to one of the groups at random (sealed envelopes) by a research assistant who is blinded from the intervention groups. Then, the next participant will be allocated to the other, and the process continues.

### 2.4 Interventions

The interventions in this study will be conducted for 12 weeks. The Pulsed ultrasound-added physiotherapy group will receive low-intensity pulsed ultrasound and conventional physiotherapy, while the control group will receive conventional physiotherapy alone. Pulsed ultrasound-added physiotherapy and control groups will receive conventional physiotherapy interventions 4 to 5 times for the first-week post-TKA and 2 to 3 times a week for a further 11 weeks. Besides, the application of low-intensity pulsed ultrasound (4–5 times for the first-week after surgery and 2–3 times a week for a further 2 weeks) in the pulsed ultrasound-added physiotherapy group will be started at post-operative day 2.

The conventional physiotherapy that will be used in this study is based on the therapy program for TKA implemented in the Universiti Kebangsaan Malaysia Medical Centre and also based on evidences which showed that exercise interventions which include stretching, strengthening,^[34]^ and functional exercises provide better outcome in recovery following TKA.^[35–37]^ The rehabilitation program for TKA is shown in Table 1.

**Table 1:**
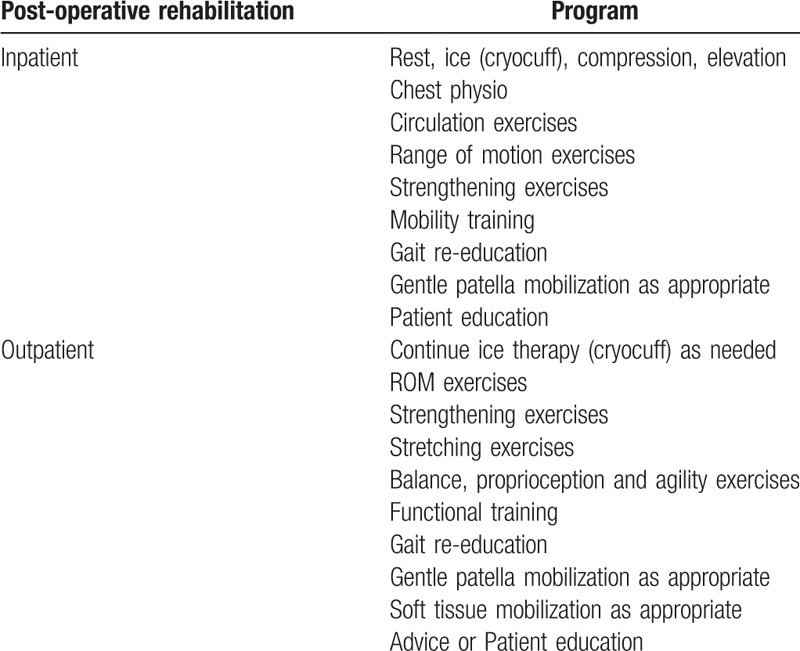
Rehabilitation program for TKA (Universiti Kebangsaan Malaysia Medical Center).

Low-intensity pulsed ultrasound (GYMNA Pulson 330, Netherlands) will be administrated for 5 minutes with the pulse ratio of 1:4 (20% duty cycle); 3 MHz frequency; and the intensity will be determined based on the thickness of the individual patient's tissues. The designated surgeon will measure the thickness of tissues using patella caliper during the surgical procedure. The area to be treated is the medial aspect of the knee which is about 1 cm away from the medial border of the patella. Transducer head and any equipment related to the pulsed ultrasound treatment will be cleaned with alcohol-based swab before and after the application.

All participants will be instructed to do home exercise program 2 times per day. The home exercise program is adopted from an exercises program for knee surgery used by physiotherapy in the university medical center (Table 2). Once-per-week telephone call will be attempted for each participant to remind them to do the home exercises program and to monitor their progression and potential adverse effects related to exercise such as cramps and muscle soreness. Exercises diary will be provided to all participants to record their home exercises practice.

**Table 2:**
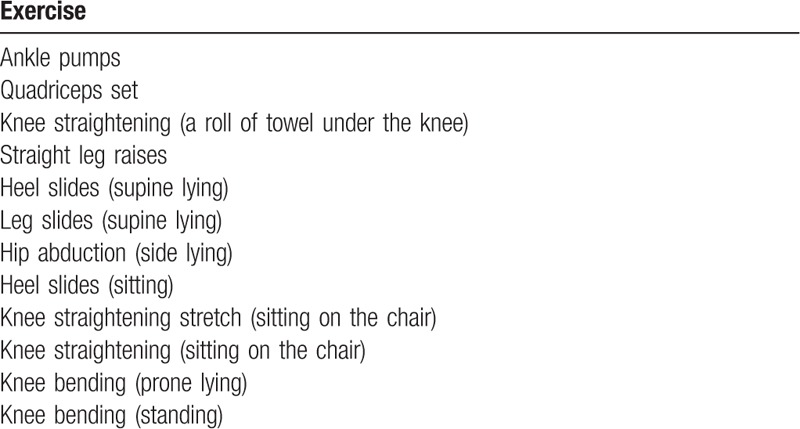
Home exercise program (Universiti Kebangsaan Malaysia Medical Center).

The treating physiotherapists and participants are not blinded to the group allocations and interventions. The practicality of this process will also be tested in the pilot study. The pilot study is conducted for 3 weeks and all participants of both groups in the pilot study are treated 4 to 5 times for the first-week post-TKA and 2 to 3 times a week for a further 2 weeks. Any amendments to the procedure and process of the pilot study will be reported and justified before implementation into the main study.

### 2.5 Outcomes

The primary outcomes (physical impairments) are as follows:

1. Pain will be measured using a 10 cm visual analogue scale (VAS) which 0 cm for “no pain” and 10 cm for “unbearable pain.” The VAS has been shown as a valid and reliable measure for assessing pain after TKA.^[38]^
2. Knee swelling will be determined by measuring the knee circumference (1 cm proximal to the base of the patella) using a measuring tape. The value nearest 0.1 cm will be recorded. This outcome measure has excellent reliability on patients with TKA, with the smallest detectable difference of 1 cm.^[39]^
3. A standard long-arm goniometer (EZ Read, JAMAR) will be used to identify any limitation of active knee range of motion with the participant positioned in supine. The participant will be asked to actively slide his/her heels on the bed to do knee flexion and extension, and the maximal active knee flexion and extension will be measured. The reliability of active knee range of motion measurement using goniometer was established in patients with TKA.^[39]^
4. Quadriceps strength will be measured isometrically using a hand-held dynamometer (microFET 2, HOGGAN Scientific, LLC). The hand-held dynamometer had been used to assess change in quadriceps strength of patients with TKA in the previous studies.^[20,21]^ The measurement will be taken with the participant in supine position and the knee in 30° flexion (a roll of the towel will be placed under the knee). The hand-held dynamometer force pad will be placed proximal to the ankle joint. The participant will be asked to push against the dynamometer force pad as maximum as possible. The participants will perform two trials for 5 s hold per each trial and 30 s of rest between the trials. The knee extensor strength will be recorded in kilograms force which the higher peak force achieved between 2 trials will be used.^[20]^

The secondary outcomes are as follows:

1. Functional performance measures
  - Timed up and go (TUG) measures the time taken by a participant to complete rising from a chair, walking 3 m, and turning, walking back to the chair and sitting. The armed chair (seat height of 47 cm) will be used. The participant will be instructed to walk safely as quickly as possible. This test measures patients’ mobility with excellent reliability on patients following TKA.^[40]^
  - 11-step stair test will also be used to assess functional performance of the participant. This test will use a staircase with eleven steps (17 cm height steps with a depth of 30 cm and 110 cm width) with handrails on both sides, and a platform at the top and one at the bottom (110 cm by 140 cm). The participant will start the test with climbing up the stairs, turn around on the top platform, and climbing down the stairs, as fast as possible. The participants will be allowed to use 1 handrail if required. The time to complete the test will be recorded. This test has good reliability on the patient with TKA in measuring the physical performance of the patient.^[41]^
  - Six-minute walk test will be used to measure how far the participants could walk in 6 min. The participants will be instructed to walk as far as possible on a level surface in 6 min and allowed to use an assistive device if necessary. The assessor will use a measuring wheel to record total distance covered. The participants could also rest for a while during the test as needed. This test has an excellent reliability in assessing functional performance of patients with TKA.^[42]^
2. Quality of life will be assessed using the 5-level EQ-5D. This outcome measure has two parts which are the EQ-5D descriptive system and the EQ visual analogue scale (EQ VAS). The EQ-5D descriptive system consists of 5 dimensions which are mobility, self-care, usual activities, pain/discomfort and anxiety/depression. Each dimension has 5 levels of health state (level 1: no problems, level 2: slight problem, level 3: moderate problems, level 4: severe problems, and level 5: extreme problems). Besides, the EQ VAS has a vertical VAS which will be used by the participants to rate their health. The scale is numbered from 0 (worst health) to 100 (best health). The EQ-5D was used as the generic health measure for patients with TKA in previous studies.^[43,44]^ Besides, the Malays version of EQ-5D was shown can be used in Malaysia with the acceptable psychometric properties.^[45]^

### 2.6 Assessment of outcomes

Two days prior to TKA surgery, a researcher will verbally invite potential participants and briefly explain the purpose and procedure of the study before distributing the information sheets to them. At post-operative day 2, the participants will be further screened for eligibility and an informed consent will be obtained from patients who are eligible and agreed to participate. Once recruited, the socio-demography, information of height, weight and body mass index (BMI) of the participants will be recorded. The dose of analgesic medication used will be obtained from patients’ medical record (in-patient) and based on the patients’ report (out-patient).

All participants will undergo baseline assessments before allocated alternately into the two groups at post-operative day 2 for physical impairments, and quality of life following removal of drainage tubes and discontinuation of patient-control analgesia. Baseline assessment for functional performance will be performed at week 1 after the surgery.

Assessment of outcomes following intervention will be conducted at 4 assessment time points which are at week 1, 4, and 12 following TKA surgery and at 3-month follow-up (6 months after the surgery). Outcomes on physical impairments will be assessed at the end of week 1, 4, and 12 following the surgery and at 3-month follow-up. Whereas, outcome on the functional performance will be assessed at week 4 and 12 after the surgery and at 3-month follow-up. Finally, the interventions’ outcome on quality of life of the participants will be assessed at 3-month follow-up or 6-month post-surgery.

A trained physiotherapist who is blinded to the participants’ group allocation is responsible to conduct and record all assessments. To avoid recall bias, the recorded measurements of patient's pre-intervention status will not be accessible to others during the patient's post-intervention assessment.

### 2.7 Sample size

The sample size required for this study will be calculated based on the results of an ongoing pilot study. The pilot study involves 10 subjects per group; a sample size which is considered adequate to assess the effect size in a pilot study of clinical trial.^[46]^ The approximates of parameters, mainly effect size, obtained from the pilot study will then be inputted in G∗Power 3.1. ANOVA repeated measures, within-between interaction statistical test, with the number of groups and measurements 2 and 5 respectively will be used in the calculation. Level of significance is set at an alpha level of 0.05 and a statistical power of 80% will be chosen.

### 2.8 Data analysis

All data will be analyzed by the researcher using the statistical package for the social sciences (SPSS) 20.0 software, IBM Corporation. If missing data are identified, intention-to-treat will be used to ensure unbiased estimates of treatment effects between the two groups, and all participants who are recruited at baseline will be included in the outcome analysis.^[47]^

The characteristics of the participants will be presented in descriptive statistics. Mixed model ANOVA will be used to evaluate time, group and interaction effects of the interventions on participants’ pain, knee swelling, knee range of motion and quadriceps strength, as well as on the patients’ functional performance and quality of life. Further, multiple regression will be used to determine how much do recovery in physical impairments following low-intensity pulsed ultrasound as an adjunct to conventional physiotherapy influence the functional performance and quality of life of the patients. The level of significance is set as *P* < .05.

### 2.9 Ethics and dissemination

The study received ethical approval from Research Ethics Committee of the Universiti Kebangsaan Malaysia Medical Centre (study code NN-2018-111). Each participant can withdraw anytime during the study without giving any explanation. All personal information and data provided by participants will be kept confidential. Investigators in this study will have access to the final trial dataset. The findings of this study will be published in a peer-reviewed journal.

## 3 Discussion

This study will compare the changes in physical impairments between patients with TKA who received low-intensity pulsed ultrasound as an adjunct to conventional physiotherapy and patients who received conventional physiotherapy only. Previous study^[31]^ did not specifically look at the effect of the interventions on the recovery of other important physical impairments such as pain, swelling and quadriceps weakness after TKA. Therefore, this study will provide knowledge regarding the effects of adding low-intensity pulsed ultrasound in post-operative rehabilitation into conventional physiotherapy on recovery of more physical impairments which are pain, swelling, knee range of motion, and quadriceps strength of patients with TKA.

Further, results of the secondary outcomes will provide information on the recovery of the functional performance and quality of life between patients with TKA who received low-intensity pulsed ultrasound and patients who did not receive the treatment. Well-targeted post-operative rehabilitation intervention can further improve the patients’ recovery, and eventually enhance the patients’ functional performance and quality of life. A study stated that pain and lower limbs function are important predictors for the improvement in quality of life of patients after TKA.^[48]^ Results of this study will be further analyzed to assess the influence of the recovery of physical impairments following pulsed ultrasound-added conventional physiotherapy on functional performance and quality of life of patients with TKA.

It is our hope to be able to determine the benefits of adding low-intensity pulsed ultrasound into conventional physiotherapy in enhancing the recovery of physical functions in patients with TKA. Findings from this study can therefore be used as a reference for designing future studies and in establishing evidence regarding the use of low-intensity pulsed ultrasound on population after TKA surgery.

## Acknowledgments

We thank Efri Noor Muhammad Hendri, physiotherapist in the university medical center who shared the information on rehabilitation program and home exercise program for TKA patients at the center.

## Author contributions

Study conceptualization and design: Munayati Munajat, Nor Azlin Mohd Nordin, Nor Hamdan Mohamad Yahya, Ahmad Hafiz Zulkifly

**Conceptualization:** Nor Azlin Mohd Nordin, Munayati Munajat, Nor Hamdan Mohamad Yahya, Ahmad Hafiz Zulkifly.

**Methodology:** Nor Azlin Mohd Nordin, Munayati Munajat, Nor Hamdan Mohamad Yahya, Ahmad Hafiz Zulkifly.

**Writing – original draft:** Munayati Munajat.

**Writing – review & editing:** Nor Azlin Mohd Nordin, Munayati Munajat.
